# Digital Health Equity and COVID-19: The Innovation Curve Cannot Reinforce the Social Gradient of Health

**DOI:** 10.2196/19361

**Published:** 2020-06-02

**Authors:** Allison Crawford, Eva Serhal

**Affiliations:** 1 Virtual Mental Health and Outreach Centre for Addiction and Mental Health Toronto, ON Canada; 2 Department of Psychiatry University of Toronto Toronto, ON Canada

**Keywords:** virtual health, digital determinants of health, digital health equity, digital health, equity, COVID-19, public health, eHealth, social

## Abstract

Digital health innovations have been rapidly implemented and scaled to provide solutions to health delivery challenges posed by the coronavirus disease (COVID-19) pandemic. This has provided people with ongoing access to vital health services while minimizing their potential exposure to infection and allowing them to maintain social distancing. However, these solutions may have unintended consequences for health equity. Poverty, lack of access to digital health, poor engagement with digital health for some communities, and barriers to digital health literacy are some factors that can contribute to poor health outcomes. We present the Digital Health Equity Framework, which can be used to consider health equity factors. Along with person-centered care, digital health equity should be incorporated into health provider training and should be championed at the individual, institutional, and social levels. Important future directions will be to develop measurement-based approaches to digital health equity and to use these findings to further validate and refine this model.

## Introduction

The public health crisis posed by coronavirus disease (COVID-19) has ignited rapid implementation of digital health care. In this commentary, we urge the implementation of health equity–informed digital health solutions. We introduce the Digital Health Equity Framework (DHEF) to identify the digital determinants of health and their links to digital health equity. In the current response to the COVID-19 pandemic, digital health has been rightly heralded as an innovative health solution that can ensure ongoing access to clinical care and allow public health measures that stem rapid viral transmission and spread [1,2]. However, unexamined inequities in access to and implementation of digital health as well as the quality of care afforded by digital health can recapitulate and deepen the inequities that have long existed within our health care system.

Digital health is broadly defined as “the field of knowledge and practice associated with the development and use of digital technologies to improve health*”* [3] across the full range of health technologies introduced into care, including telehealth, mobile health apps and wearable technologies, and online health services and tools. During the COVID-19 public health crisis, two modes of digital health have been commonly used: virtual health care, or televideo-enabled interactions between health providers and patients, and health information that is accessed online or via mobile apps.

Some media commentators [4] have stated that COVID-19 is the “great leveler” because it knows no boundaries and can infect rich and poor, young and old. However, this uncritical perspective misses the systemic factors that impact outcomes of illness and create health inequities between communities and across the life courses of individuals. Mounting evidence suggests that the COVID-19 pandemic has far greater associated morbidity and mortality in racialized groups that struggle with poverty and poor access to health care; the pandemic has also been suggested to compound preexisting inequities [5]. Similarly, there has been a lack of attention to health equity in the development of digital health solutions [6]; therefore, when these solutions are applied within the pandemic response, they may have unintended consequences of furthering health inequity. For example, access to technology can be limited by poverty, under-resourcing of health systems and neighborhoods, homelessness, and other factors that decrease engagement with technology and with digital health literacy skills. Health providers may also lack training and competencies in consideration of digital health equity as well as the cultural humility to understand how their patients and communities may experience or interact with technology. Digital health technologies interact with social, cultural, and economic realities and with social determinants of health to indirectly contribute to health equity.

## The Digital Health Equity Framework (DHEF)

**Figure 1:**
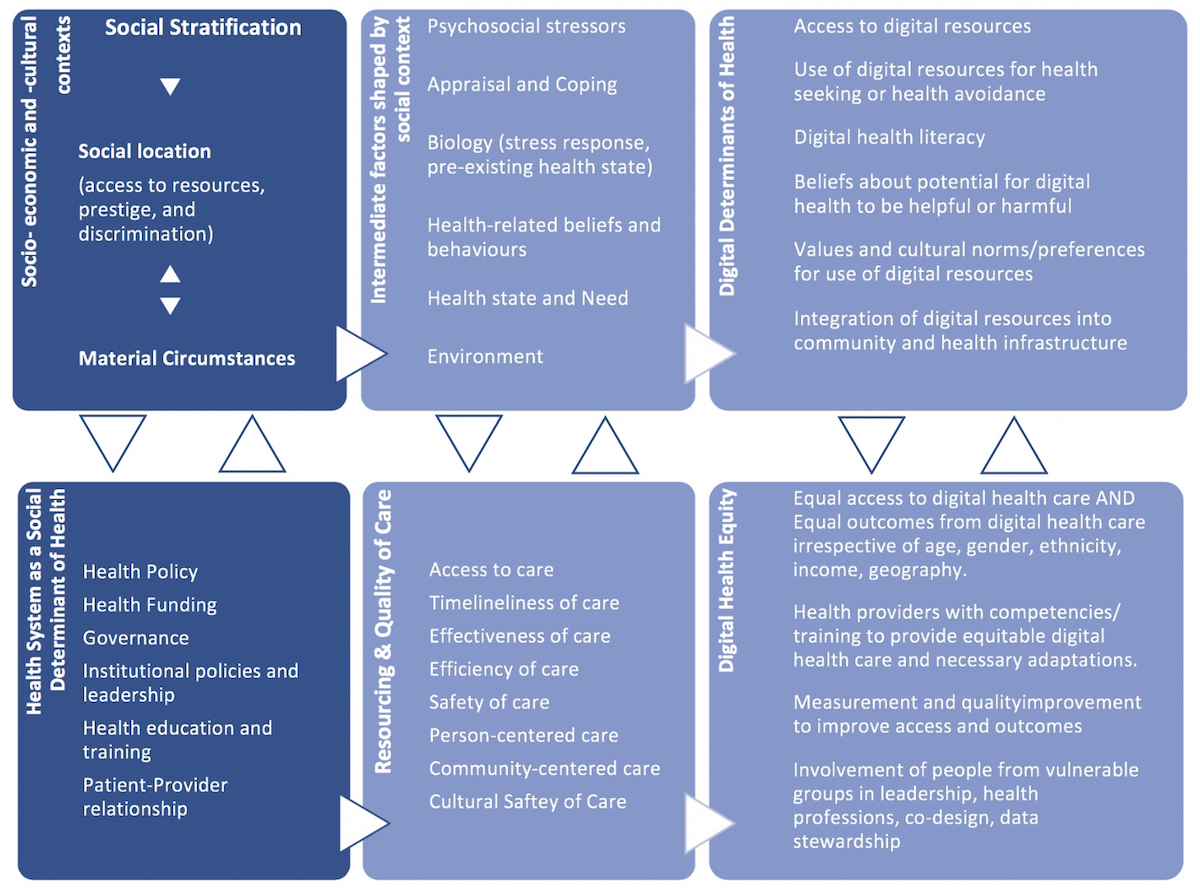
Digital Health Equity Framework.

Here, we propose the DHEF (Figure 1), which applies many health equity factors outlined by Dover and Belon [7] in 2019, integrated with digital determinants of health and digital health equity. In their recent approach, Dover and Belon survey the many frameworks proposed for considering social determinants of health and address some of the limitations of the health equity field by moving from a superficial description of factors to a more comprehensive, ecological approach that considers the multitude of social, cultural, and economic factors that impact health and well-being as well as the interactions among these factors. Most significantly, they link these social determinants to health equity and begin to delineate a structure through which health equity can be measured within organizations and at a health system level.

In Dover and Belon’s model, which informs the foundation of the DHEF, the process of social stratification within economic and cultural social contexts refers to the hierarchical allocation and unequal distribution of power, prestige, and resources; this stratification assigns individuals to a social location, which is defined by intersectional factors such as race, age, income, geography, rurality, gender, ability, and occupation as well as other social factors. A person’s social location governs exposure to health-related risks and vulnerabilities, including discrimination. A person’s social location and material circumstances can be mutually reinforcing, and they also intersect with intermediate factors that shape health and health behaviors, including psychosocial stressors; styles of appraisal and coping; biology, including current and health status and preexisting conditions; health-related beliefs and behaviors; current health needs; and their environment [7].

Similarly, in the DHEF (Figure 1), *digital determinants of health* interact with other *intermediate health factors*, such as psychosocial stressors, preexisting health conditions, health-related beliefs and behaviors, and the environment, along with the person’s current health state and needs. For example, access to digital health resources and digital health literacy interact with the degree and kind of psychosocial stress a person is currently experiencing; job loss or poverty, level of education, and previous exposure to digital media can all impact access. Styles of coping and appraisal of risk, along with health-related beliefs, can shape beliefs and behaviors regarding digital health; for example, some patients may have a tendency to avoid health care or to minimize risk, leading to issues such as corollary avoidance of digital health care, privacy-related concerns, or failure to appraise the quality of digital health information. Just as a person’s environment shapes their health care access and quality, it also shapes their digital health access and quality; people living in overcrowded homes may lack privacy or, as with underhoused and homeless populations, may not have access to digital health solutions at all. All these intermediate factors are set into play, are reinforced, and in turn reinforce the *socioeconomic and sociocultural context* and the *social stratification* process. Intersections of race, gender, and geography are among the variables that determine one’s power in society and define one’s social location, which is closely linked to and interacts with one’s material circumstances.

The DHEF expands on the *health system as a social determinant of health*. Moving the dial on health equity, including digital health equity, requires looking beyond individual factors to the health system. We need to ensure that at every level, from health care providers to institutions, insurers, health regulators, and government, we are able to detect, understand, and work to improve the *resourcing and quality of digital health care* for all social groups to reduce digital health disparities. Quality of care, which ensures that care is person-centered, safe, timely, effective, and efficient, is also care that is equitable [8]. This includes the quality of digital health care. For example, if digital health care is not experienced as culturally safe by a population of users, or if the environments (living spaces, communities, institutions, and infrastructure) and material circumstances of groups of people are not considered when developing institutional digital health strategies or in the provisioning of funding and remuneration models for providers working with vulnerable populations, the quality of digital care will suffer, and digital health equity will be impacted. The DHEF model highlights the importance of approaching digital health technologies from an ecological perspective, considering the ways that the use of technology by an individual extend out into (and are shaped by) their social, cultural, and economic position in the world. The case shown in Textbox 1 illustrates this interplay using COVID-19 as an example.

**Example of digital health equity related to COVID-19.**

Mr Seow is a 48-year-old man living with his older adult father and three children in an apartment in a socioeconomically disadvantaged urban neighborhood. His economic insecurity forces him to continue in his job as a food delivery worker; this exposes both Mr Seow and his father to higher risk of COVID-19 infection. Overcrowding in their apartment adds to that risk. Mr Seow has poorly controlled diabetes, a preexisting health condition that creates a risk of worse health outcomes with COVID-19 infection and is a consequence of the same social inequities. He also suffers from depression.

Mr Seow’s health risks related to COVID-19 may be further compounded by his own style of appraising risk and his own health behaviours. He tends to minimize risk, he smokes, and he has limited awareness of public health advice. His community has aging infrastructure, and the health resources in his neighborhood include an overcrowded hospital with inadequate access to lifesaving equipment such as ventilators.

The effects of these social, cultural, and economic factors compound across Mr Seow’s life course and include intergenerational cumulative effects of social location and material circumstances. Mr Seow’s father, an older adult, is an immigrant who spent time in a detention center many years ago; he equates hospital with imprisonment, also avoids health care, and has many unmet health needs.

The promise of digital health in relation to Mr Seow’s situation is evident. Virtual visits (ie, telehealth) will allow him to continue to access health care for his diabetes and depression while enabling him to avoid exposure to the overcrowded hospital in his community. This has potential to mitigate some of his health risks and support health equity. However, digital determinants of health and likely outcomes of digital health equity should also be examined. Mr Seow’s outcome of receiving virtual health may vary greatly compared with that of a man of the same age living in more economically advantaged circumstances. These inequities must be recognized to be addressed. For example, does Mr Seow have access to technology that supports virtual care? Can he meet with a health provider in a private space within his crowded living situation? Does he have a minimum degree of digital health literacy, with the ability to access and appraise reliable information? Do his own personal beliefs and values about technology support his use of digital health? Are digital health resources available in his community, and do they integrate with other points of access to health care in his community (ie, is this option covered by his insurer, or is it integrated with his primary care provider)?

At the provider level, are Mr Seow’s health care providers, such as doctors and nurses, trained to think about health equity? Do they possess the cultural humility to recognize some of these potential gaps and disadvantages of virtual care for Mr Seow and to make necessary adaptations? Are they trained to monitor outcomes of virtual health in their setting and to consider sociocultural variables in these outcomes?

Addressing health equity extends far beyond the individual and the patient-provider dyad to systemic and social contexts. To ensure digital health equity, primary care, hospitals, and governments must have digital health strategies that identify and addresses potential gaps in digital health care based on these digital determinants of health. To know whether they are successful in achieving digital health equity, they need to conduct ongoing measurements of equity and of digital health outcomes. Health equity considerations must also be part of quality of care considerations, along with person-centered care that considers patient choice and autonomy.

There are examples, albeit few, of digital health research that incorporates considerations of social determinants and health equity, particularly in developing contexts [6]; however, this approach needs to become mainstream in all implementations of digital health. Only one week ago, the World Health Organization (WHO) released its 4-year draft global strategy on digital health [3], which aims to support international efforts “to develop the infrastructure for information and communication technologies for health…[and] to promote equitable, affordable and universal access to their benefits” along with promoting the development of national digital health strategies. In line with the 2030 Agenda for Sustainable Development, the WHO seizes on the “great potential to accelerate human progress, to bridge the digital divide and to develop knowledge societies.” Within the WHO strategy, one of the strategic objectives is to advocate for people-centered health systems that are enabled by digital health. For example, they seek to advance “digital health literacy, gender equality and women’s empowerment and inclusive approaches to adoption and management of digital health technologies.” The report mentions a number of approaches that relate to health equity throughout; however, these are as yet unformulated within the WHO’s implementation plan.

To consider digital health equity within our health and social contexts, we need to establish systematic ways to ensure that potential health inequities are identified and addressed in digital health policies, strategies, and programs so that existing health inequities are not reinscribed onto our virtual health landscapes. Implementation science, which specifies factors relevant to increased uptake of an innovation, is emerging as a critical factor to guide the spread and scaling of digital health; however, implementation models often fail to incorporate health equity factors or to address social determinants of health. To ensure health equity within digital health, we need to be purposeful in implementing digital health in an equitable way and in measuring health outcomes through an equity lens. If we do not collect health equity data, we cannot monitor health equity outcomes. In turn, understanding the population health needs of vulnerable groups can identify barriers to implementation that create innovation gaps, such as the gap between population health interest in digital health care and the capacity of US hospitals to deliver digital health care in response to COVID-19 [9]. Integrating a health equity approach such as the DHEF with a health implementation approach is an urgent need, particularly at this time of rapid advances in digital health innovation. This should include determining appropriate health equity metrics and measures for digital health.

Perhaps most importantly, to avoid duplicating the social stratification that exists in society at large, we need to ensure the meaningful involvement of people from marginalized and vulnerable groups in positions of digital health leadership, as health providers, and in codesign at all stages of innovation and implementation, including as stewards of their own health outcome data. In times of crisis, such as the current COVID-19 pandemic, utilitarian principles of innovation are often viewed as a way to maximize overall social benefits, while egalitarian principles that address inequalities are set aside [10].

## Conclusion

There is no question that virtual health care can provide sustained access to essential health care; however, this commentary aims to draw attention to the unintended health equity impacts of the pivot to digital health care from the early days of the response to the current pandemic. One of the major limitations of this commentary is that we do not have available data to quantify these concerns. We believe that measurement-based approaches to health equity are a high priority for digital health research. There is emerging work in this area [11], and we hope that the framework we propose can further stimulate investigation into the multiple ways in which digital determinants of health may impact digital health equity. In turn, these data will lead to refinement of the proposed framework. During the COVID-19 pandemic and in its aftermath, we need to urge ongoing attention to health equity, including digital health equity, and to develop processes and measures to prevent our own blind spots and inattention in this regard. The celebrated curve of innovation cannot reinforce the social gradient of health, whereby people in less advantaged socioeconomic positions have less access to digital health care, poorer quality of digital health care, or worse health outcomes.

## Abbreviations

COVID-19: coronavirus disease
DHEF: Digital Health Equity Framework
WHO: World Health Organization

## References

[1] Keesara S, Jonas A, Schulman K (2020). Covid-19 and Health Care's Digital Revolution. N Engl J Med.

[2] Torous J, Jän Myrick Keris, Rauseo-Ricupero N, Firth J (2020). Digital Mental Health and COVID-19: Using Technology Today to Accelerate the Curve on Access and Quality Tomorrow. JMIR Ment Health.

[3] (2020). World Health Organization.

[4] Dooc E (2020). Business Mirror.

[5] Williams DR, Cooper LA (2020). COVID-19 and Health Equity-A New Kind of "Herd Immunity". JAMA.

[6] Sinha C, Schryer-Roy A (2018). Digital health, gender and health equity: invisible imperatives. J Public Health (Oxf).

[7] Dover DC, Belon AP (2019). The health equity measurement framework: a comprehensive model to measure social inequities in health. Int J Equity Health.

[8] Institute of Medicine, Committee on Quality of Health Care in America (2001). Crossing the Quality Chasm: A New Health System for the 21st Century.

[9] Hong Y, Lawrence J, Williams D, Mainous I (2020). Population-Level Interest and Telehealth Capacity of US Hospitals in Response to COVID-19: Cross-Sectional Analysis of Google Search and National Hospital Survey Data. JMIR Public Health Surveill.

[10] Emanuel EJ, Persad G, Upshur R, Thome B, Parker M, Glickman A, Zhang C, Boyle C, Smith M, Phillips JP (2020). Fair Allocation of Scarce Medical Resources in the Time of Covid-19. N Engl J Med.

[11] Were MC, Sinha C, Catalani C (2019). A systematic approach to equity assessment for digital health interventions: case example of mobile personal health records. J Am Med Inform Assoc.

